# Diffuse idiopathic pulmonary neuroendocrine cell hyperplasia (DIPNECH) in association with an adenocarcinoma: a case report

**DOI:** 10.1186/1752-1947-2-21

**Published:** 2008-01-25

**Authors:** Arne Warth, Esther Herpel, Astrid Schmähl, Konstantina Storz, Philipp A Schnabel

**Affiliations:** 1 Institute of Pathology, University Hospital Heidelberg, Heidelberg, Germany; 2 Department of Radiology, Thoraxklinik Heidelberg, University of Heidelberg, Germany; 3 Department of Thoracic Surgery, Thoraxklinik Heidelberg, University of Heidelberg, Germany

## Abstract

**Introduction:**

Diffuse idiopathic pulmonary neuroendocrine cell hyperplasia (DIPNECH) is a rare disorder and information on this disease is limited, especially with regard to its management and prognosis. It has become generally accepted that DIPNECH is a precursor lesion to pulmonary carcinoid tumors.

**Case presentation:**

Here we report on a 60-year-old female patient with DIPNECH and an associated pulmonary adenocarcinoma.

**Conclusion:**

This case contributes to a better understanding of the disorder and its associated pathologies.

## Introduction

Diffuse idiopathic pulmonary neuroendocrine cell hyperplasia (DIPNECH) is an exceedingly rare disorder and only 40 cases have been described in the literature to date [1]. According to the current WHO classification, this disorder is characterized by one of the following: a generalized proliferation of scattered single cells, small nodules or linear proliferations of pulmonary neuroendocrine cells [2]; in addition, it is considered to be a precursor for pulmonary carcinoid tumors.

## Case presentation

**Figure 1:**
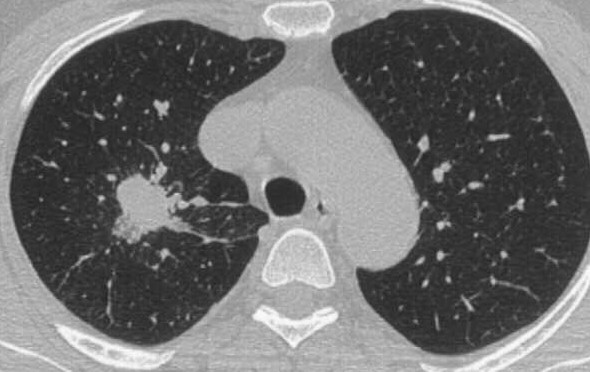
Preoperative CT scans. The preoperative CT scans clearly demonstrate the main tumor in the upper lobe of the right lung.

**Figure 2:**
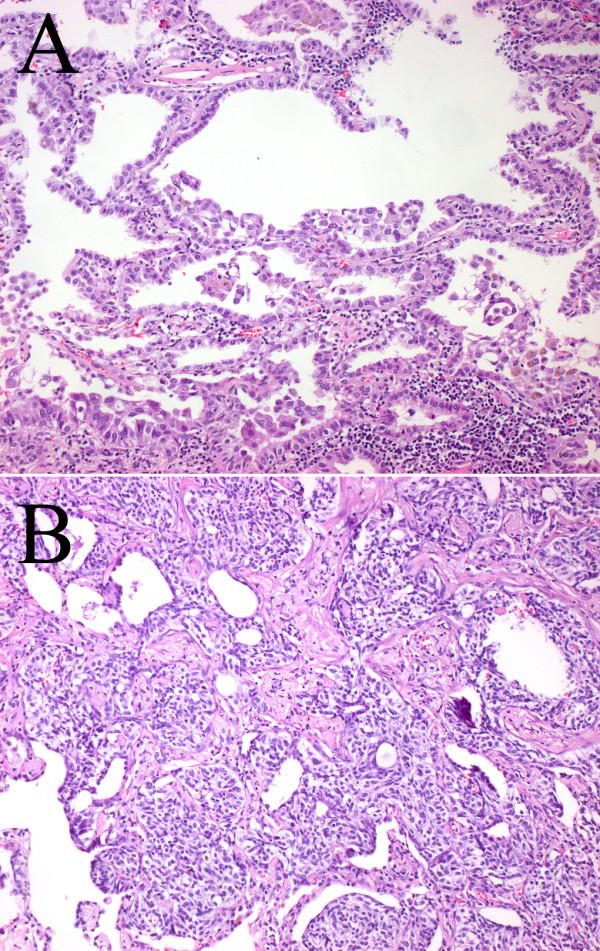
Histology of the adenocarcinoma and a representative tumorlet. Pathological processing of the resected specimens revealed an adenocarcinoma of a mixed subtype with partial neuroendocrine differentiation (A; primary magnification ×10) and multiple tumorlets, i.e. diffuse idiopathic pulmonary neuroendocrine cell hyperplasia (DIPNECH; B; primary magnification ×10).

Here we report on a patient with DIPNECH who coincidently developed a pulmonary adenocarcinoma. The 60-year-old female patient was initially referred to our hospital because computed tomography scans revealed a tumor-like lesion measuring 2.9 cm in its widest diameter in segment 2 (right upper lobe, posterior segment) of the right lung. Additionally, several lesions as large as 0.6 cm were evident in segments 4 (right middle lobe, lateral segment) and 6 (right lower lobe, superior segment) of the right lung. These lesions were suggested to represent metastases of the lesion in segment 2. A CT scan of the chest (Fig. 1) was indicated following detection of a pulmonary nodule in the right upper field on routine chest -x-ray. The patient had a 10 pack-year smoking history and complained of shortness of breath upon admission; the remaining review of symptoms was negative. Pre-operative diagnostics revealed arterial hypertension and moderate left ventricular hypertrophy and pulmonary function tests were unsuspicious (VC 143%, FEV1 128%). Since the CT findings raised the suspicion of a malignancy, a diagnostic thoracotomy with a concurrent sleeve lobectomy of the right upper lobe was performed in combination with a systematic lymphadenectomy. Pathological processing of the specimens revealed a 3.5 × 3 × 2.8 cm adenocarcinoma of a mixed subtype with partial neuroendocrine differentiation (Fig. 2). The tumor was strongly positive for CK7, CK18, TTF1 and SPA and focally positive for CEA, NSE and chromogranin A. The proliferation rate (Ki67) was 20–30%. Besides this main tumor there were multiple small metastases with a similar degree of differentiation in the upper right lobe as well as in segment 4 of the middle right lobe. Tumor infiltration of intrapulmonary and mediastinal lymph nodes was also present. Therefore, the TNM classification for the pulmonary adenocarcinoma was pT4, pN2 (16/27), pM1, G3. However, further processing of the multiple small lesions in the upper and middle lobe revealed five foci less than 5 mm in diameter with a different trabecular and nest-like morphology (Fig. 2). In these lesions, the cells were strongly positive for CD56, synaptophysin, NSE and chromogranin A and focally positive for CK7, CK18, TTF1 with a proliferation rate (Ki67) of 1–2%. Therefore, the diagnosis of multiple tumorlets (microcarcinoids) was made. Due to the multicentricity of the lesions and a size of <5 mm in diameter, the correct diagnosis was DIPNECH. Lesions >5 mm are classified as carcinoids according to the current WHO classification[2]. There were no clinical symptoms suggestive of any proteins and/or hormones released, but interestingly, NSE (20 ng/ml) and CEA (33 ng/ml) were slightly elevated, whereas Cyfra was within the normal range (1.4 ng/ml). The postoperative course of the patient was uneventful. Six months after the operation the patient is still alive and no tumor recurrence has been detected so far.

DIPNECH is an exceedingly rare disease involving generalized proliferation of pulmonary neuroendocrine cells, which leads to an occlusion of the bronchial lumina and consequent clinical symptoms such as shortness of breath. Due to its neuroendocrine origin, its similar morphology to pulmonary carcinoids and particularly due to its association with pulmonary carcinoids, the disease is considered to be a precursor lesion for theses entities. Only 40 cases of DIPNECH have been reported in the literature to date [1] and there are no predictive histological or genetic data available so far. However, it has become generally accepted that DIPNECH is a precursor to pulmonary carcinoid tumors [3]. In a recent study including 1090 patients, who received resections for primary lung tumors, Ruffini and colleagues found that the overall prevalence of pre-invasive lesions for lung carcinomas was 6.7%. Only 3 of these 1090 cases were associated with DIPNECH and the primary tumors were carcinoids in all of these cases [4]. We have recently reported a similar case in which DIPNECH was associated with a carcinoid [5]. The current report represents the first case of a patient with DIPNECH accompanied by a pulmonary adenocarcinoma of mixed subtype with partial neuroendocrine differentiation. The adenocarcinoma was positive for typical markers such as CK7, CK18, TTF1, and SPA and additionally, it was positive for NSE and CEA, which were also measured to be elevated in the patient's serum. Interestingly, besides typical carcinoid markers such as CD56, synaptophysin, and chromogranin A, the DIPNECH lesions were also positive for NSE. However, it remains elusive if the elevated NSE levels in the patient's serum belong to the adenocarcinomas, the DIPNECH lesions or a combination of both. Nevertheless, our findings raise the hypothesis of a common pathogenic background of pulmonary tumors with neuroendocrine differentiation, which should further be investigated. Although it is unlikely that DIPNECH is a precursor lesion for other tumors of the lung with neuroendocrine differentiation besides carcinoid tumors [6], this possibility cannot yet be excluded considering the small number of the cases described to date. Since it has been suggested that DIPNECH represents an underrecognized spectrum of disease and since it is being increasingly diagnosed [7], we report this case to contribute to a better understanding of the disorder and its associated pathologies. However, the association of DIPNECH with a higher overall cancer incidence should be regarded carefully, since there is evidence that malignancies at other sites lead to an increased use of imaging and thereby to a more frequent detection of DIPNECH [7]. Therefore, more data are needed to accurately draw a conclusion on the incidence of associated malignancies.

## Conclusion

DIPNECH is a rare disorder and it is considered to be a precursor lesion for pulmonary carcinoid tumors. Information on the disease is still limited, especially with regard to management and prognosis. This case is the first report of a patient with DIPNECH in association with a pulmonary adenocarcinoma. Since an increasing incidence of DIPNECH cases has been noted in the past few years, we report this case to contribute to a better understanding of the disorder and its associated pathologies.

## Abbreviations

DIPNECH = diffuse idiopathic neuroendocrine cell hyperplasia; CK7 = cytokeratin; CK18 = cytokeratin 18; TTF1 = thyroid transcription factor 1; SPA = surfactant protein A; CEA = carcinoembryonic antigen; NSE = neuron specific enolase.

## Competing interests

The author(s) declare that they have no competing interests.

## Authors' contributions

AW wrote the manuscript. EH diagnosed the specimens and collected data. AS performed and diagnosed the CT scans. KS performed the operation, pre- and post-operative patient management. PAS diagnosed the specimens and made final corrections of the manuscript. All authors read and approved the final manuscript.

## Consent

Written consent was obtained from the patient for the publication of the report.
